# Violent Partners or a Specific Class of Offenders? A Criminal Career Approach to Understanding Men Involved in Intimate Partner Sexual Violence

**DOI:** 10.1177/10790632231224356

**Published:** 2023-12-27

**Authors:** Julien Chopin, Francis Fortin, Sarah Paquette, Jean-Pierre Guay, Olivier Péloquin, Eric Chartrand

**Affiliations:** 1 University of Lausanne, Lausanne, Switzerland; 2 4440 Laval University, Quebec, QC, Canada; 3 1763 Simon Fraser University, Burnaby, BC, Canada; 4 5622 University of Montreal, Montreal, QC, Canada; 5 Provincial Sex Offender Coordination Division, Sûreté du Québec, Montreal, QC, Canada; 6 University of Portsmouth, Portsmouth, UK

**Keywords:** intimate partner sexual violence, criminal career, comparison, criminal trajectories

## Abstract

The current study investigates the criminal career of individuals involved in intimate partner sexual violence (IPSV). Specifically, the goal is to determine whether men who engage in IPSV can be distinguished from those who engage in intimate partner non sexual violence (IPNSV) only and whether criminal trajectories in the resulting subgroup are heterogeneous. The sample comes from a Canadian database including a total of 12,458 individuals involved in IPSV and 32,474 individuals involved in IPNSV). Bivariate and multivariate analyses are performed to examine the differences in the two groups while latent profile analysis allows examining the heterogeneity of characteristics of men who engaged in IPSV. Findings indicate that the criminal career of men who engage in IPSV follows a pattern that is clearly distinct from that of men who engage in IPNSV only and is more specialized in terms of sexual offenses. Results also show that the criminal trajectories followed by the men who engage in IPSV are heterogeneous. Four profiles of different trajectories were identified. Both theoretical and practical implications are discussed.

## Introduction

Intimate partner sexual violence (IPSV) is one of the most underestimated and misunderstood forms of violence committed in an intimate-partner context (Bagwell-Gray et al., 2015; Black et al., 2011). This specific form of intimate partner violence (IPV) has been the subject of specific studies, but knowledge on the topic is limited due to the difficulty in identifying cases and establishing measurements for the phenomenon (Logan et al., 2007, 2015). IPSV can be defined as any unwanted, non-consensual sexual activity or behavior imposed by one partner upon another within the context of an intimate relationship. This can encompass a wide range of behaviors, including but not limited to sexual assault, rape, coercion, sexual harassment, and any form of sexual activity carried out without the clear and voluntary consent of one partner by the other in the context of an intimate relationship (Breiding et al., 2015; World Health Organization, 2012). Studies examining the prevalence of IPSV have estimated that it may account for one-quarter of all sexual assaults (Bachman & Saltzman, 1995), or between 7 and 13% of the total incidence of violent events (Bagwell-Gray et al., 2015; Basile, 2002; Black et al., 2011; Tjaden & Thoennes, 2006). Although it is very difficult to accurately determine the extent of this phenomenon, it is clear that it is poses major social, security, and public health problems. Previous studies have shown that IPSV has serious consequences for victims in terms of declining mental health (Bagwell-Gray, 2021; McFarlane et al., 2005; Messing et al., 2014) as well as increased substance abuse (McFarlane et al., 2005; Ullman & Sigurvinsdottir, 2015), suicide risk (Cavanaugh et al., 2011; Puri et al., 2011; Weaver et al., 2007), and decreased sexual and physical health (Logan et al., 2007; Seyller et al., 2016; Tjaden, 2000) (see Barker et al., 2019 for a comprehensive literature review). Indirect victimization is also an important problem: approximately 20% of the children of partners involved in such violence have witnessed acts of IPSV and are at increased risk of developing high levels of anxiety as well as difficulties related to social withdrawal (Campbell & Alford, 1989; Graham-Bermann et al., 2006; Symes et al., 2014). Understanding and addressing these issues is crucial for preventing and managing recidivism in cases of IPSV. The criminal career paradigm provides a useful framework for analyzing the criminal trajectories of individuals who commit IPSV to better understand who they are, the risks they pose, and their needs. This paradigm can be defined as the longitudinal pattern of an individual's involvement in criminal activities over time (Blumstein et al., 1986; DeLisi & Piquero, 2011; Piquero et al., 2003). Studies of those whose criminal careers involve IPV have rarely distinguished between individuals involved in IPSV and those who do not commit sexual violence, hence considering them as a homogeneous group. However, studies of extrafamilial interpersonal violence have shown that men who engage in sexual offenses are very different from those who display other violent behaviors, including homicide (Beauregard & DeLisi, 2018, 2021; Beauregard et al., 2018; DeLisi & Beauregard, 2018). Identifying differences, if any, between those who commit IPV with no sexual component and those who commit IPSV is crucial in developing prevention and management strategies and programs for treatment and reinsertion. Are the most appropriate programs for individuals who engage in IPSV those that target violence in general or those that deal specifically with sexual violence? This study builds on recent studies that suggest that analyzing criminal trajectories makes it possible to distinguish men who engage in IPSV from those who engage in intimate partner non-sexual violence (IPNSV) (Jung et al., 2021; Léveillée et al., 2022). Previous studies, while significant initial steps, relied only on basic indicators of criminal careers, which limits development of a comprehensive understanding of these phenomena. The objective of this study is therefore to analyze the criminal careers of men who engage in IPSV to determine if they constitute a specific type of offender.

## Intimate Partner Violence and the Criminal Career Framework

The criminal career paradigm was elaborated by Blumstein et al. (1986) as a way to improve the study of the criminal trajectory of individuals by systematizing its components. A criminal career was defined as the temporal sequence of criminal events in the life of an individual, from first criminal act to last (Kyvsgaard, 2002; Piquero et al., 2003). This paradigm focuses on several key dimensions, including onset, duration, escalation, specialization, and desistance. Onset refers to when an individual first engages in criminal behavior. Duration involves the length of time an individual remains involved in criminal activities. Escalation refers to whether an individual's criminal behavior becomes more severe or frequent over time. Specialization pertains to whether an individual becomes specialized in certain types of crimes. Finally, desistance is the process of an individual ceasing their criminal activities or significantly reducing their involvement (MacLeod et al., 2012; Piquero et al., 2007; Zara & Farrington, 2016). The objective was not to explain a particular criminal act but to identify and organize the elements in individual criminal trajectories to make it possible to identify specific patterns (DeLisi & Piquero, 2011). The criminal career was operationalized in terms of four areas: participation (number and types of offenses committed during a career), frequency (number of offenses committed over a particular period), seriousness (severity of the criminal behaviors), and duration (various temporal characteristics of the criminal career) (Blumstein et al., 1986).

Previous studies have looked at IPV in relation to each of these dimensions. Investigation of the level of specialization of men who engage in IPV showed that, in general, these individuals were generalists whose polymorphic criminal careers were characterized by an important variety in the kinds crimes committed (Dowling et al., 2021; Hilton & Eke, 2016; Theobald et al., 2016). Research also showed that a criminal career characterized by a high number of offenses is more likely to include IPV offenses (Piquero et al., 2014) and that there was an association between higher numbers of IPV offenses and intimate partner homicide (Kivisto, 2015; Messing et al., 2017; Snider et al., 2009). Studies of the severity of the criminal careers of men who engage in IPV found that criminal careers with high severity indicators often include IPV offenses and recidivism (Jones & Gondolf, 2002; Ménard et al., 2009; Murphy et al., 1998; Woodin & O'Leary, 2006). It should be noted, however, that several studies found no significant relationship between criminal career severity and IPV offenses (Coley et al., 2016; Cunha et al., 2022; Goldstein et al., 2016; Rempel et al., 2008; Robinson, 2017). These differences could be related to the heterogeneity of the methods used to operationalize the severity of a criminal career (i.e., injury severity, severity calculated according to an index). Finally, the study of the length of the criminal career of individuals involved in IPV showed an early onset of delinquency with a tendency toward longer criminal careers and earlier commission of first crime than those whose crimes do not involve IPV offenses (Dowling et al., 2021; Loinaz, 2014; Wooldredge, 2007).

There have been few studies focusing on the specific criminal careers of men who engage in IPSV and the studies that do exist have focused on general indicators of frequency. In a study of a sample of 103 men involved in IPV (76 in IPNSV and 27 in IPSV), Léveillée et al. (2022) found that men who engage in IPSV were more likely to have a criminal history than those involved in IPNSV only. Men who engaged in IPSV were also more likely to be involved in economic violence or a combination of economic, verbal, physical, and psychological violence. Interestingly, these results differ from those in a study by Jung et al. (2021) which compared a group of 145 men who engaged in IPSV with 145 men who engaged in IPNSV only, 145 men who were involved in sexual violence against known (extrafamilial context), and 145 men who were involved in sexual violence against unknown victims. Their results indicated that men who engaged in IPSV were less likely to have a criminal history and less likely to recidivate than those who engaged in IPNSV. However, men who engaged in IPSV were more likely to have a criminal history and a higher risk of recidivism than men with sexual offenses in an extra-familial context (against both known and stranger victims) (Jung et al., 2021).

## Heterogeneity Among Men Who Engage in Intimate Partner Sexual Violence

Several studies have used a variety of classifications to examine the heterogeneity of men who engage in IPSV. These typologies are often based on patterns of aggression or motivation. Finkelhor and Yllo (1985), who developed the first of such typologies, propose using three categories based on information from interviews with 50 victims of marital rape. This classification distinguishes *battering rape* (hits, humiliation), in which the individual wants to punish his partner; *force only rape* (the minimal amount of force needed to achieve victim compliance is used), in which individuals demand satisfaction from their partners, who are seen as their possession; and *obsessive rape* (mutilation, spanking, humiliation, sexual sadism), in which individuals inflict pain to achieve sexual pleasure (Finkelhor & Yllo, 1985). A second typology, proposed by Russell (1990) and based on interviews with 87 IPSV victims, distinguishes three distinct categories based on individuals’ attitudes: (1) a preference for sexually assaulting a partner rather than maintaining a consensual relationships (sadism, physical and psychological violence), (2) maintaining both consensual and non-consensual relationships with partners in order to satisfy sexual desires (sense of entitlement, use of force to achieve their goals), (3) a preference for consensual relationship with a partner but also able to have non-consensual relationships to satisfy sexual desires (Russell, 1990). More recently, Proulx and Beauregard (2014) proposed a typology, based on a sample of 43 cases, that focuses on analysis of pathways to sexual aggression. Their results suggest a three-group typology: *angry* (motivated by anger, have high levels of expressive aggression, dissatisfaction with their sexual lives, social isolation, and several internal and external problems)*, hypersexual* (high levels of violence, deviant sexual fantasies, high frequency of sexual contacts, and external problems), and *lonely* (limited use of physical violence and individuals with low self-esteem, social isolation, hostility towards women and hypersexuality) (Proulx & Beauregard, 2014). The most recent typology, proposed by Bagwell-Gray (2021) and based on interviews with 28 female victims, identifies four groups, related to the type of sexual activity (penetrative act vs. non-penetrative act) and the type of force used (non-physical force vs. physical force): *intimate partner sexual assault* (unwanted penetrative sexual acts combined with physical force or the threat of such force), *intimate partner sexual coercion* (low levels of physical force combined with highly invasive sexual acts), *intimate partner sexual abuse* (non-physical and noninvasive strategies used to exhibit control over women sexually), and *intimate partner forced sexual activity* (high levels of physical violence and non-penetrative sexual acts).

## Aim of the Study

The literature review revealed that studies of the criminal careers of individuals involved in IPSV are few and limited. It appears that men who engage in IPSV, like men who engage in sexual offenses against extrafamilial victims, may differ from those who do not sexually assault their partners, although researchers often fail to make this distinction, dealing with those involved in IPV as a homogeneous group. Studies that distinguish these two groups have reported potential differences in their criminal trajectories, although these results are exploratory and inconsistent. While analysis of the literature is fundamental to improving our understanding of this understudied phenomenon, it reveals several limitations in previous studies. First, sample sizes are very limited and could prejudice the reliability of results. Second, the indicators used to operationalize the criminal history are elementary (e.g., the presence or absence of a criminal history) and do not demonstrate a global understanding of the criminal career paradigm.

A more in-depth understanding of the criminal career of individuals involved in IPSV is important on several levels. From a theoretical perspective, criminal career analysis offers valuable insights into individual behavior and interactions with the justice system, enhancing our understanding of criminal behavior. From a practical perspective, the criminal career pattern provides information on the risks of recidivism, the type of management required, and the effectiveness of sanctions and could be important for various criminal justice system professionals. The objective of the present study is therefore to provide an in-depth study of the criminal careers of men who engage in IPSV, comparing the indicators of the criminal careers of men who engage in IPSV with those who engage only in IPNSV to determine if the criminal trajectories of men who engage in IPSV are heterogeneous. In this context, based on the existing literature, we formulated two hypotheses:

H_1_: Men who engage in IPSV will have a specific type of criminal career that differs from the criminal career trajectories of men who engage only in IPNSV.
H_2_: Men who engage in IPSV will have heterogeneous criminal career trajectories.

## Method

### Data and Sample

The data used in this study were taken from a database that contains information on all IPV crimes reported to the authorities in the province of Quebec, Canada, and includes information on 125,616 individuals whose criminal careers began between January 1, 1990, and May 25, 2022. This database, known as the General Index of Police Information Module (General Index/MIP), is an integral part of the Quebec Police Information Center (CRPQ) and was established in 1980 to enhance information retrieval efficiency, ultimately aiding in crime resolution. It functions as a computerized police database, obliging all police services in Quebec to input data from police investigation files. The data are aggregated by a team of crime analysts from the Sureté du Québec who are specialized in interpersonal violence. Information pertaining to criminal careers and the corresponding characteristics of crimes is aggregated to individuals who are made unique through the use of specific identifiers. The system mandates the recording of essential information related to the crime, including details about the circumstances (e.g., location, weapon used, suspect's actions, duration of actions, commencement and conclusion times, reporting time), the suspect (e.g., age, gender, physical characteristics, place of residence), the victim(s) (e.g., age, gender, physical characteristics, place of residence), the nature of the relationship between suspect(s) and victim(s), the investigation start and end dates, and specific attributes of the crime under investigation, such as the suspect's intoxication status or the use of various weapon types. Notably, the dataset contains no missing data due to the system's mandatory data logging requirements for all criminal events. The database extraction encompasses all individuals involved in reported cases of IPV reported to judicial authorities in the province of Quebec from 1966 to 2022. IPV, is defined as the range of psychological, physical violent acts as well as property damage occurring between partners or former partners and that cause physical, sexual and/or psychological harm (Guay et al., 2021; Oram et al., 2022; White et al., 2023). The relationship between intimate partners is defined as a close and intimate connection between two individuals. This relationship can take various forms, including marriage, cohabitation, boyfriend-girlfriend relationships, dating relationship, or any other form of relationship where there exists an emotional or affectionate bond between the partners. This database has already been used in several previous research (Chopin et al., 2023).

In using this data, we made several methodological choices aimed at increasing the comparability of identified groups. First, we included only individuals who had committed at least one act of physical violence against a partner, excluding those whose crimes involved fraud or psychological violence. Second, we included only cases involving male offenders. Using these criteria reduced the possibility that we were comparing heterogeneous groups, thus decreasing statistical variability (see Beauregard & Martineau, 2017; Dobash et al., 2007). These decisions resulted in a sample of 49,985 individuals, 39.79% of the total database.

The sample of individuals selected included two mutually exclusive subgroups. First, we selected all individuals involved in at least one sexual assault against a partner or ex-partner, a subgroup of 12,458 individuals or 9.92% of the sample. In this study, sexual assault is defined as one partner not having consented to sexual contact, the legal definition in Canada. The operationalization was carried out based on the legal definition based by the presence of charges against an individual. Sexual contact can include kissing, fondling, or sexual intercourse. Events in the database include several types of sexual offending: aggravated sexual assault (injures, mutilates, disfigures, or endangers the life of the complainant), sexual assault with a weapon (threatens or inflicts bodily harm), sexual assault (assault committed under circumstances of a sexual nature such that the victim’s sexual integrity is violated). We also included assaults that cause little or no bodily harm to the victim, such as non-contact sexual assault (e.g., pressure of a sexual nature, masturbation in front of the victim without her consent), voyeurism, and non-consensual intimate image distribution. It is important to note that the identification of the sexual nature of the assaults relies on official data, and these data may undergo reclassification by judicial authorities during the legal process. While there is a high level of confidence in categorizing cases due to data recording procedures and information triangulation, it is not uncommon for certain cases of sexual assault to be reclassified as non sexual for various reasons (e.g., lack of evidence, victim retractions, etc.). Second, we selected all men who had been involved in violence against a partner or ex-partner but whose criminal career trajectory did not include sexual assault against an intimate partner. This subgroup included 32,474 individuals. In order to compare homogeneous case groups, we made several methodological choices. These choices were made to reduce data variability and isolate the effects of the variables of interest (i.e., to avoid statistical noise) (see Creswell & Creswell, 2017). First, we decided to include only cases involving male perpetrators. This decision was based on previous research indicating different dynamics depending on the gender of the perpetrator, both in the context of sexual assaults (Williams & Bierie, 2015) and IPV (Mackay et al., 2018). Second, we chose to consider only female victims. This choice was also made based on previous studies suggesting significant differences in assault dynamics for same-sex couples (Blosnich & Bossarte, 2009; Rollè et al., 2018).

The average age of men in the sample at the time of first offense was 32.6 years (*SD* = 12.49). The average length of criminal career was 2796.41 days (*SD* = 2483.92), with an average of 9.64 (*SD* = 12.77) criminal events. During their criminal careers these men were involved, on average, in 2.03 (*SD* = 1.82) instances of IPV and 7.51 (*SD* = 11.81) criminal events in a non-domestic context.

Looking specifically at subgroups, men who engaged in IPSV had an average age of 29.41 years (*SD* = 11.87) at the time of their first offense. The average length of their criminal career was 2524.95 days (*SD* = 2634.21), with an average of 8.29 (*SD* = 12.29) criminal events. Men who engaged in IPNSV only had an average age of 34.17 years (*SD* = 12.60) at the time of their first offense, an average length of criminal career of 2666.05 days (*SD* = 2356.93), and an average number of 10.10 (*SD* = 13.24) criminal events.

### Measures

The different variables described in this section were used in both binary logistic regression and latent profile analysis (LPA), described in the section on analytical strategy below.

#### Dependent Variable

We used one dichotomous dependent variable in this study, which made it possible to divide the sample into two subgroups: men with sexual offenses in their criminal trajectory and men without sexual offenses in their criminal trajectory.

#### Independent Variables

In order to operationalize individual criminal careers, we used the different dimensions suggested by Blumstein et al. (1986), using a total of 12 continuous variables to describe participation, frequency, severity, and duration of the criminal career. To operationalize participation, we used eight variables that describe the kind and number of offenses committed by each individual during his criminal career: (1) number of homicides [ $\bar{X}$ = .02; *SD* = .16], (2) number of violent offenses [ $\bar{X}$ = .10; *SD* = .37], (3) number of property offenses [ $\bar{X}$ = 2.01; *SD* = 5.54], (4) number of liberty violation offenses (i.e., these offenses encompass a range of actions that infringe upon an individual's freedom and rights. Some examples of offenses unlawful confinement, kidnapping, false imprisonment) [ $\bar{X}$ = 1.98; *SD* = 2.35], (5) number of breaches of conditions [ $\bar{X}$ = 2.65; *SD* = 5.66], (6) number of drug offenses [ $\bar{X}$ = .80; *SD* = 2.07], (7) number of sexual offenses (in a non-IPV context only) [ $\bar{X}$ = .20; *SD* = .74], (8) number of other offenses [ $\bar{X}$ = .81; *SD* = 1.77]. We also used a variable to describe (9) the variety of offenses (the number of different types of offenses) [ $\bar{X}$ = 3.77; *SD* = 2.84]. To operationalize frequency, we used (10) the general lambda measure (average number of offenses per year) [ $\bar{X}$ = 1.45; *SD* = 1.25]. To operationalize severity, we used (11) the average gravity score (calculated using the Statistics Canada crime severity index, see Wallace 2009) [ $\bar{X}$ = 113.72; *SD* = 115.84]. Finally, to measure the temporal aspects of the criminal career, we used (12) age at first criminal event [ $\bar{X}$ = 32.62; *SD* = 12.44]. This variable pertains to the age at which the first criminal event, as reported to the authorities, occurred.

To perform the LPA, we utilized the key variables of criminal participation described above, which were divided based on context (i.e., IPV or non-IPV context). The variables included in the model are as follows: number of homicides (IPV and non-IPV context), number of sexual offenses (non-IPV context), number of violent offenses (IPV and non-IPV context), number of condition breaches (IPV and non-IPV context), number of property offenses (IPV and non-IPV context), number of drug offenses (non-IPV context).

To test the external validity of the LPA model, we used 13 additional continuous variables which describe aspects of sexual victimization within a domestic setting and the characteristics of the victimization context.

#### Sexual Victimization in a Domestic Context

To better explore the different types of sexual victimization, we used six continuous variables that describe the different sexual offenses reported to authorities: (13) number of aggravated sexual assaults [ $\bar{X}$ = .00; *SD* = .08], (14) number of sexual assaults with a weapon [ $\bar{X}$ = .01; *SD* = .08], (15) number of sexual assaults, excluding those in categories 13 and 14 [ $\bar{X}$ = .23; *SD* = .45], (16) number of non-contact sexual assaults [ $\bar{X}$ = .00; *SD* = .01], (17) number of voyeurism events [ $\bar{X}$ = .00; *SD* = .05], (18) number of non-consensual intimate image distribution events [ $\bar{X}$ = .01; *SD* = .11].

#### Victimization Context

To characterize the general context of victimization we used the following six variables: (19) number of different victims in an IPV context (partner or ex-partner) [ $\bar{X}$ = 2.13; *SD* = 2.07], (20) number of victims in a relationship context (as opposed to ex-relationship) [ $\bar{X}$ = 1.18; *SD* = 1.25], (21) number of events involving an individual with a mental disorder [ $\bar{X}$ = .43; *SD* = 1.48], (22) number of criminal events without injuries in an IPV context [ $\bar{X}$ = 1.36; *SD* = 1.26], (23) number of criminal events with minor injuries in an IPV context [ $\bar{X}$ = .57; *SD* = 1.07], (24) number of criminal events with serious injuries in an IPV context [ $\bar{X}$ = .02; *SD* = .15].

### Analytical Strategy

This study used a three-step analytical process. The first step was assessment of the differences between men who engage in IPSV and those who engage in IPNSV at the bivariate level (Mann–Whitney *U* test^1^) for the set of independent criminal career variables. The second stage involved analyzing these differences at the multivariate level and computing a binary logistic regression using only the significant variables (*p* ≤ .05) from the bivariate analyses. The goal was to create a multivariate model based on the specific characteristics associated with men who engage in IPSV. This analysis provided the final and best model. Multicollinearity was checked for the variables included in the multivariate analyses and no VIFs above 2.56 or tolerance below .39 were found (see Supplemental Table 1). For the binary logistic regression model, we chose to keep the original samples and compared the 32,474 men who engaged in IPNSV to the 12,458 men who engaged in IPSV. This choice was made in order to capture as precisely as possible the characteristics of the groups in our sample. To ensure that the differences in the size of the two groups did not influence the results of the analyses, we compared a randomized sample (*n* = 13,000) from the group of men who engaged in IPNSV to the sample of men who engaged in IPSV (*n* = 12,458). The results showed minor differences in the Beta values and no difference in the significance levels. To better analyze the significance of the results, we have included the 5% confidence intervals in the tables.

The last analytical step consisted of examining the heterogeneity of characteristics of men who engaged in IPSV criminal trajectories and victimization correlates. LPA was conducted using Latent Gold V6.0 software package to test heterogeneity. LPA statistical procedure is used to identify heterogeneity that is not directly observable or measurable in order to detect underlying patterns in a set of data or subgroups of individuals who share important behavioral characteristics (Collins & Lanza, 2010). Seven models were computed (Supplemental Table 2) and the Bayesian Information Criterion (BIC) and Akaike Information Criterion (AIC) were used to evaluate the model fit and identify the number of classes to use in LPA. We used additional victimization context variables to test the external validity of the model and improve its depth. Bivariate analyses (Kruskal–Wallis test^2^) were used to identify significant differences between the different profiles. Such a procedure is commonly used with LPA (Brownfield & Sorenson, 1987; Vaughn et al., 2008) and makes it possible to distinguish whether the differences identified in the main classification model, which are based on a limited number of indicators, are also found for other indicators in the phenomenon being studied. The authors takes responsibility for the integrity of the data, the accuracy of the data analyses, and have made every effort to avoid inflating statistically significant results. We report how we determined our sample size, all data exclusions, all manipulations, and all measures in the study.

### Ethics

This study received ethical approval from the ethics committee of the University of Montreal (2022–102-D).

## Results

Table 1 presents the bivariate analysis between men who engaged in IPSV and those who engaged in IPNSV only for criminal career indicators. Findings show that there are significant differences between these subgroups in the number of homicides, liberty violation offenses, violent offenses, property offenses, conditions breaches), drug offenses, sexual offenses in a non-domestic context, and other offenses characterizing criminal career participation. For the variety indicator, men whose records did not show sexual offenses had a higher average score than those with sexual offenses. Findings suggest that men without sexual offenses were more likely to have a higher general lambda score than those with sexual offenses. The latter were more likely to have a higher average gravity score and to be younger at the time of their first criminal event known to the autorities.

**Table 1.** Bivariate Analysis of Criminal Career Indicators Predictive of Type of Offender (*N* = 44,932).

	General Sample *N* = 49,952		IPNSV Offender (*n* = 37,494)		IPSV Offender (*n* = 12,458)				
	$\bar{X}$	*SD*	$\bar{X}$	*SD*	$\bar{X}$	*SD*	Mann-Whitney U	η^2^	*p* Value
Participation									
Number of homicides	.02	.16	.02	.17	.02	.15	239,106,946.00	.000	.013
Number of liberty violation offences	.10	.37	.10	.37	.09	.37	238,064,459.00	.000	.005
Number of violent offences	1.98	2.35	2.31	2.33	1.02	2.15	98,554,662.50	.057	<.001
Number of property offences	2.01	5.54	2.18	5.84	1.53	4.52	211,056,880.50	.003	<.001
Number of condition breaches	2.65	5.66	2.85	5.81	2.07	5.14	206,173,117.00	.004	<.001
Number of drug offences	.80	2.07	.87	2.16	.60	1.74	221,790,620.00	.003	<.001
Number of sexual offences (non-domestic context)	.20	.74	.16	.58	.30	1.07	226,857,227.00	.007	<.001
Number of other offences	1.03	2.10	1.11	2.17	.81	1.85	215,219,007.00	.004	<.001
Variety	3.77	2.84	3.87	2.81	3.48	2.92	209,315,046.50	.004	<.001
Frequency									
Lambda	1.45	1.25	1.47	1.26	1.40	1.23	231,818,943.50	.001	<.001
Severity									
Average gravity score	113.72	115.84	96.88	100.52	163.59	141.11	109,424,531.00	.063	<.001
Career length									
Age at first criminal event	32.62	12.44	33.72	12.45	29.41	11.87	187,897,446.50	.023	<.001

Notes. ***p ≤ .001.

Table 2 presents the findings of the binary logistic regression of factors predictive of inclusion in intimate partner sex offender category for men who engaged in IPSV. This model presents an area under the curve (AUC) of .84 (see Supplemental Table 3), a Cox and Snell *R*^2^ of .21, and a Nagelkerke *R*^2^ of .31. Results show that men who engaged in IPSV were less likely to perpetrate homicides, liberty violation offenses, violent offenses (β = −.64, *p* < .001), property offenses, drug offenses, and other offenses than men who engaged in IPNSV only. Men who committed sexual offenses were also less likely to have a higher variety score than those who had not committed such offenses. Both the general lambda and the average gravity score were likely to be higher for men who engaged in IPSV, who were also likely to begin their criminal career at a younger age at the time of their first criminal event known to the autorities.

**Table 2.** Binary Logistic Regression of Criminal Career Factors Predictive of Inclusion in Intimate Partner Sex Offender Category (*N* = 44,932).

	B	S.E.	Wald Test	Exp (B)	CI [95%]	
Number of homicides	−3.18	.15	463.42	.04***	.03	.06
Number of liberty violation offences	−.38	.04	94.24	.68***	.63	.74
Number of violent offences	−.64	.01	2863.87	.53***	.52	.54
Number of property offences	−.03	.00	60.70	.97***	.96	.98
Number of condition breaches	.09	.00	560.25	1.10***	1.09	1.11
Number of drug offences	−.06	.01	47.39	.95***	.93	.96
Number of sexual offences (non-domestic context)	.19	.02	124.03	1.21***	1.17	1.25
Number of other offences	−.01	.01	.84	.99	.98	1.01
Variety	−.06	.04	184.78	.94***	.94	.95
Lambda	.05	.01	24.96	1.06***	1.03	1.08
Average gravity score	.01	.00	2902.23	1.01***	1.01	1.01
Age at first criminal event	−.03	.00	1046.49	.97***	.96	.97
Constant	−.90	.05	272.59	.41***		
X^2^	11,971.88					
−2 log likelihood	44,691.97					
Cox and snell *R*^2^	.21					
Nagelkerke *R*^2^	.31					
AUC	.84					
AUC CI [95%]	.84–.85					

Notes. ***p ≤ .001.

**Figure 1.**
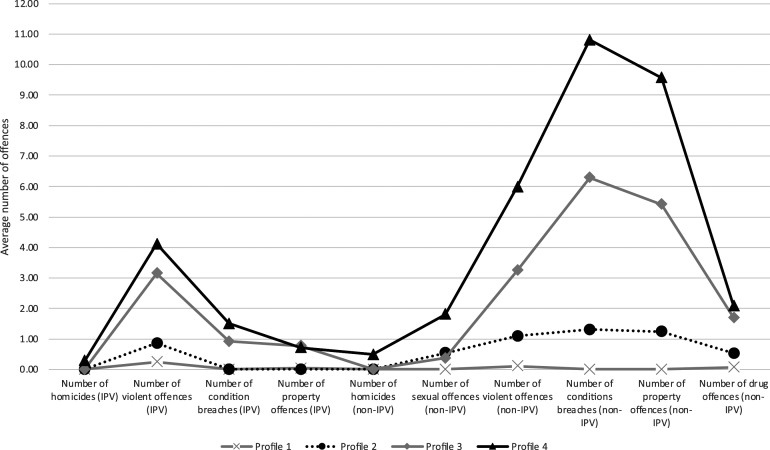
Four latent profile model - mean score of intimate partner sex offender’s criminal carrier based on class membership.

Table 3 and Figure 1 presents the findings of the LPA. To examine heterogeneity among those in the sample whose criminal trajectories included IPSV, one-to-seven-profile solutions were analyzed to determine the best LPA solution (see Supplemental Table 2), which showed that the 4-profile solution had the best fit according to BIC (−400,429) and adjusted BIC (−400,848.83). BIC and adjusted BIC decreased up to profile 4 but increased from profile 5 on. A smaller BIC suggests that the trade-off between fit and parsimony has been achieved. Entropy for the 4-profile solution was high (.99), suggesting that the predictors used are appropriate to classify the cases and that classes were highly distinct (Schwartz, 1978). The Vuong-Lo-Mendell-Rubin adjusted likelihood ratio test indicates that the 4-profile model was a significant improvement on the fit of the 3-profile model. The largest profile was the first, which included 5487 individuals (44.04% of the group of men who engaged in IPSV), while the smallest profile was the fourth, which included 277 individuals (2.22% of the group of men who engaged in IPSV).

**Table 3.** Four Latent Profile Models - Mean Score of IPSVOs’ Criminal Career Based on Profile Membership (*N* = 12,458).

	Profil 1	Profil 2	Profil 3	Profil 4
*n* =	44.04%	34.75%	18.98%	2.22%
Sample %	5487	4329	2365	277
Intimate partner context				
Number of homicides	.00	.00	.00	.29
Number of violent offences	.25	.86	3.15	4.12
Number of condition breaches	.00	.00	.92	1.51
Number of property offences	.04	.00	.77	.72
Non-intimate partner context				
Number of homicides	.00	.00	.00	.49
Number of sexual offences	.00	.54	.37	1.81
Number of violent offences	.11	1.10	5.41	5.99
Number of conditions breaches	.00	1.31	6.29	10.81
Number of property offences	.00	1.24	1.25	9.57
Number of drug offences	.07	.53	1.70	2.09

Profile 1 was labeled *Men who engage in IPSV with no other criminal offense in their trajectory*. The criminal career of individuals in this group involved only IPSV. Means for all types of offense studies approximate zero, both for offenses committed in an intimate partner context and those committed in a non-intimate partner context, although the number of violent offenses is slightly higher, with an average of .25.

Profile 2 was labeled as *Men who engage in IPSV with low numbers of non-IPV criminal activities in their trajectory*. Individuals in this group are characterized mainly by a low number of offenses: an average number of 1.10 violence offenses, 1.31 condition breaches, and 1.24 property offenses, all perpetrated in a non-IPV context.

Profile 3 was labeled *Men who engage in IPSV and display* a *medium level of offenses in a polymorphous criminal trajectory*. The criminal career of these individuals is characterized by an average number of 3.15 violent offenses in an IPV context. They were also characterized by an average number of 5.41 violent offenses, 6.29 condition breaches, and 1.25 property offenses, all in a non-IPV context.

Profile 4 was labeled *Men who engage in IPSV and display a high volume polymorphous criminal trajectory*. The criminal trajectories of these individuals are characterized by an average number of 4.12 violent offenses and 1.51 condition breaches in an IPV context. They were also characterized by an average of 1.81 sexual offenses, 5.99 violent offenses, 10.81 condition breaches, and 9.57 property offenses, all perpetrated in a non-IPV context.

Table 4 presents the findings of the external validity analysis using additional victimization indicators. Findings show that the four-profile solution external validity is satisfactory, as 11 of the 12 variables tested show significant differences between different profiles. Specifically, findings show that there are significant differences between the four profiles for the number of aggravated sexual assaults, sexual assaults with a weapon, sexual assaults (excluding aggravated and with a weapon) (, non-contact sexual assault, and intimate images distribution without consent. For the victimization context additional variables, analysis shows that there are differences between the four profiles for the number of different victims in an intimate partner context, the number of different victims in a relationship context, and the number of intimate-partner violence events where the individual was presumed by law enforcement to have mental health issues. Finally, there are differences between the four profiles regarding the number of events in an intimate-partner context that resulted in no physical injury, minor physical injury, and major physical injury.

**Table 4.** External Validity Analysis of the Four-Profile Typology (*N* = 12,458).

	Profil 1	Profil 2	Profil 3	Profil 4	Kruskal-Wallis H
*n* =	44.04%	34.75%	18.98%	2.22%	
Sample %	5487	4329	2365	277	
Sexual victimization in a domestic context					
Average number of aggravated sexual assaults	.01	.02	.02	.05	29370.00***
Average number of sexual assaults with a weapon	.02	.03	.03	.06	17102.00***
Average number of sexual assaults, all contexts	.86	.88	.95	.91	40806.00***
Average number of sexual assaults without contact	.05	.06	.04	.02	22491.00***
Average number of voyeurism events	.01	.01	.01	.01	.67
Average number of incidences of intimate image distribution without consent	.04	.04	.06	.02	19927.00***
Victimization context					
Number of different victims in a domestic context	1.22	1.85	4.17	5.29	4407513.00***
Number of victims in a relationship context	.55	.82	2.06	2.36	1426311.00***
Number of events involving an offender with a mental disorder	.12	.40	.79	.90	1915303.00***
Number of domestic violence criminal events without injuries	.70	1.03	2.18	2.53	1868695.00***
Number of domestic violence criminal events with minor injuries	.32	.56	1.53	2.07	1947655.00***
Number of domestic violence criminal events with serious injuries	.01	.01	.06	.17	383956.00***

Notes. ***p ≤ .001.

## Discussion

To examine the characteristics of individuals involved in sexual assaults committed in an IPV context, our analysis focused on the criminal career paradigm and its various components (Blumstein et al., 1986; DeLisi & Piquero, 2011; Piquero et al., 2003). The study was framed to determine whether men who engage in IPSV are similar to those who engage in IPNSV only or constitute a specific subgroup of individuals with different needs and risks (Sparks et al., 2020). This question has been previously addressed in studies of men who engage in extrafamilial sexual offenses (see Beauregard & DeLisi, 2018, 2021; Beauregard et al., 2018; DeLisi & Beauregard, 2018; Stefanska et al., 2016; Zara et al., 2022), and this previous work led us to the two hypotheses that guided this study: (1) Men who engage in IPSV will have a specific type of criminal career that differs from the criminal career trajectories of men who engage only in IPNSV, and (2) Men who engage in IPSV will have heterogeneous criminal career trajectories. Our findings confirmed both hypotheses.

### A Similar Context but a Distinct Criminal Career Pattern

The first hypothesis suggests that men who engage in IPSV have a different criminal history than men who engage in IPNSV only. Our results are congruent with previous exploratory studies (Jung et al., 2021; Léveillée et al., 2022) and suggest that the criminal trajectories of individuals in these groups differ at several points. Previous studies reported mixed findings, with Jung et al. (2021) finding that men who engage in IPSV were more likely to have a criminal career than men who engage in IPNSV only, while Léveillée et al. (2022) found the opposite. The results of our analyses highlight the complexity of operationalizing a criminal career, illustrating the limits of using simple and basic dichotomous indicators to characterize its presence or absence. To expand on previous analyses we introduced a set of indicators proposed by Blumstein et al. (1986). The results suggest differences between groups related to the participation concept. We observed a clear pattern in which men who engaged in IPSV generally exhibit a much less polymorphic criminal career than men who engage in IPNSV only (Dowling et al., 2021; Hilton & Eke, 2016; Theobald et al., 2016) and are less likely to have committed homicides, physical assaults, property crimes, liberty violation offenses, and drug offenses. These results confirm previous studies suggesting that men who engage in IPSV were less likely overall to have a criminal history (Jung et al., 2021; Léveillée et al., 2022). It is interesting to note that the criminal careers of men who engage in IPSV appear to follow a trajectory more like that of men who engage in extra-familial sexual offenses, but with a specialized profile (Beauregard et al., 2018; Jung et al., 2021; Sparks et al., 2020). First, we found that, in addition to the level and kind of crimes committed, the average level of offense variety among men who engaged in IPSV is also lower than that of the men who engaged in IPNSV only, reinforcing the idea that such individuals are more likely to specialize (Dowling et al., 2021; Hilton & Eke, 2016; Piquero et al., 2014; Theobald et al., 2016). Second, our results indicate that men who engaged in IPSV were more likely to also commit sexual assaults outside the IPV context. This finding strengthens the hypothesis that men who engage in IPSV may resemble men who commit extra-familial sexual offenses more than those whose IPV is non-sexual (IPNSV) and is congruent with findings of Jung et al. (2021). The general lambda is also higher among men who engaged in IPSV than for men who engaged in IPNSV only, suggesting an increased risk of recidivism (Jung et al., 2021; Léveillée et al., 2022). Our results are in line with those of the study by Sparks et al. (2020), which suggested that individuals involved in IPSV exhibited much more severe profiles/needs than those involved in IPV. Finally, we found that men who engaged in IPSV committed their first criminal act at a younger age than men who engaged in IPNSV only. Starting a criminal career at a younger age is often correlated with a longer criminal career (DeLisi, 2006; DeLisi & Piquero, 2011) and has been identified as a major factor in predicting the risk of recidivism in IPV (Loinaz, 2014; Wooldredge, 2007). Although they are younger, it should be noted that the various groups under study all exhibit a relatively high age of entry into a criminal career, as it is around 30 years old (Zara & Farrington, 2010). This is considered a late onset and implies individuals with characteristics and needs distinct from delinquent populations with an early onset (Zara & Farrington, 2010, 2013).

Our results suggest that it is not sufficient to characterize individuals only according to their risk of having or not having a criminal career and show that it is important to consider multiple indicators, which better reflect the complexity of their trajectories as well as the risks of recidivism.

### A Specific Criminal Path Characterized by a Continuum of Trajectories

The second hypothesis suggests that, despite a similar pattern of participation, the criminal trajectories of men who engaged in IPSV were otherwise heterogeneous. This hypothesis is based on previous typological studies aimed at classifying men who engage in IPSV (Bagwell-Gray, 2021; Finkelhor & Yllo, 1985; Proulx & Beauregard, 2014; Russell, 1990) and was confirmed in our study, with LPA identifying four distinct criminal patterns: men who engage in IPSV with no other criminal acts, men who engage in IPSV with low volume non-IPV criminal acts, men who engage in IPSV with medium volume polymorphic criminal acts, and men who engage in IPSV with high volume polymorphic criminal acts.

#### Men Who Engage in IPSV With no Other Criminal Acts

This trajectory, followed by 44.04% of men in our sample who engaged in IPSV, is characterized by an absence of criminal history other than IPSV. This pattern has been observed for individuals involved in intimate partner homicides (see e.g., Dobash et al., 2009), and is similar to the category of family-only batterers identified by Holtzworth-Munroe and her colleagues (Holtzworth-Munroe et al., 2000; Holtzworth-Munroe & Stuart, 1994). Individuals included in this profile do not engage in the most extreme forms of sexual violence (aggravated sexual assaults, sexual assault with a weapon). Violence is more likely to involve one victim and to result in few physical injuries. This description suggests that men who follow this trajectory are similar to those in the category of force-only-rape identified by Finkelhor and Yllo (1985), and to individuals identified by Russell (1990) who prefer consensual relationships with their partners but are able to have non-consensual relationships to satisfy their sexual desires.

#### Men Who Engage in IPSV With Low Volume Non-IPV Criminal Acts Trajectory

This trajectory includes the second-largest number of men who engage in IPSV (34.75%). Their criminal trajectory is characterized by commission of a limited amount of physical violence and incidences of breach of conditions and property crimes committed in a non-IPV context. These results echo those of Jung et al. (2021), who found that men who engage in IPSV were more likely to be involved in a combination of economic, verbal, and physical violence rather than restricting themselves to physical violence. In terms of sexual violence perpetrated in the IPV context, individuals included in this profile differed minimally from those in the trajectory of men who engage in IPSV with no other criminal career.

#### Men Who Engage in IPSV With a Medium Volume of Polymorphic Criminal Acts

Previous studies analyzing diverse samples of men who engage in IPV have identified individuals who follow this criminal trajectory, which is characterized by a combination of IPV and non-IPV offenses. (Dowling et al., 2021; Hilton & Eke, 2016; Theobald et al., 2016). In addition to IPSV, these individuals have significant average levels of physical violence committed in an IPV context as well as of physical violence, condition breaches, and property offenses perpetrated in a non-IPV context. The average number of IPSV is higher than for those in the first and second trajectories. The presence of a higher number of distinct victims suggests that these individuals are caught in a cycle of endemic violence, not directly linked to their partners. This profile is also characterized by the highest average number of criminal events involving the distribution of intimate images without the victim’s consent. While the level of such distribution is hard to measure using police data, it could suggest a diversification of methods used to gain control over the victim, whether in a relationship or ex-relationship context (Aikenhead, 2018; Bishop, 2022; Eaton et al., 2021; Lucas, 2022).

#### Men Who Engage in IPSV With High Volume Polymorphic Criminal Acts Trajectory

This trajectory is as extreme in terms of the acts involved as the prevalence of individuals included is low (2.22%). These individuals have a significant average number of all types of offenses committed in both IPV and non-IPV contexts. The IPSV committed by these individuals is the most serious, including the highest number of aggravated sexual assaults and assaults committed with a weapon. Similarly, individuals following these trajectories have the highest average number of different victims, the highest number of events involving mental health issues, and the highest number of victims with serious injuries. Given the different characteristics of this group, it appears that they may be associated with the obsessive rape category proposed by Finkelhor and Yllo (1985), the category of individuals who prefer to sexually assault their partners rather than have consensual relationships with them identified by Russell (1990), and the category of intimate partner sexual assault identified by Bagwell-Gray (2021).

## Conclusion

The objective of this study was to determine whether men who engage in IPSV can be distinguished from those who engage in IPNSV only and whether criminal trajectories in the resulting subgroup are heterogeneous. Results show that the criminal career of men who engage in IPSV follows a pattern that is clearly distinct from that of men who engage in IPNSV only and is more specialized in terms of sexual offenses, in both IPV and non-IPV contexts. Furthermore, several indicators (general lambda, age at the first criminal event known to the authorities) suggest that the risk of recidivism is greater among men who engage in IPSV. Results also show that the criminal trajectories followed by the men who engage in IPSV included in our study were heterogeneous. Four profiles of different trajectories were identified: Men who engage in IPSV with no other criminal acts, Men who engage in IPSV with low volume non-IPV criminal acts, Men who engage in IPSV with medium volume polymorphic criminal acts, and men who engage in IPSV with high volume polymorphic criminal acts. In summary, our study reveals a dual dynamic within the criminal trajectories of men engaging in IPSV: a specialized pattern indicative of shared traits, coexisting with a broader heterogeneity reflecting individual differences among IPSV offenders. The specialized pattern we observe suggests that, despite the diverse backgrounds and experiences of individuals within the IPSV group, there are discernible commonalities in their criminal trajectories. Simultaneously, the broader heterogeneity emphasizes the diversity of individuals within the IPSV group. This diversity spans a spectrum of histories of prior criminal involvement and their severity that contribute to the complexity of IPSV trajectories.

The results of this study, the first to recognize the importance of a detailed analysis of the criminal career of men who engage in IPSV, have both theoretical and practical implications. Regarding theoretical implications, the results confirmed the relevance of using the criminal career paradigm to study the trajectories of individuals to determine whether they reveal patterns that make it possible to distinguish between subgroups of offenders. Using the different concepts proposed for this paradigm made it possible to identify clear differences in criminal career patterns. The results reinforce previous suggestions that men who engage in IPSV differ from those involved in IPV and that it might be important to consider these subgroups separately in future studies. From a practical standpoint, the results have significant implications for practitioners, confirming the need for distinct management approaches for men who engage in IPSV behavior and suggesting that programs tailored toward different categories of sex offenders could prove more effective and suitable for their reintegration while minimizing the risk of reoffending. To address the question of whether the most suitable programs for individuals engaging in IPSV were those targeting general violence or sexual violence, our findings clearly favor the latter option. The results unequivocally suggest that these individuals are more akin to those involved in sexual assaults than those involved in IPNSV. In this context, it is important to be aware that these trajectories are part of a continuum, which must be taken into account in attempts to assess the level of risk. The different profiles developed in this study could be used as a complement to clinical and actuarial assessments. The identification of specific risk factors can aid law enforcement, legal professionals, and support services in recognizing individuals at a heightened risk of reoffending. Furthermore, in line with the analysis by Sparks et al. (2020), our findings confirm that IPSV offenders have specific criminogenic needs. The criminal career indicators emphasized the need for high-intensity treatment programs for individuals who pose severe risks to society due to the severity of their trajectories. Specifically, specializing in sexual assaults and early engagement in delinquency may be the two key areas of focus for practitioners to reduce recidivism and increase the likelihood of desistance. Approaches such as cognitive-behavioral therapy, the development of positive life and social skills, and trauma treatment could be further explored to enhance the reintegration of these individuals and reduce their risk of reoffending (e.g., Morgan & Gilchrist, 2010; Sparks et al., 2020; Weldon, 2016). As to the victim assistance, gaining a more profound insight into the offender’s criminal career can result in enhanced support and safety measures for victims. The discernment of behavioral patterns in offenders proves invaluable when devising safety strategies for victims. For instance, if data indicate a propensity for offenders to escalate violence over time, victims can be advised on the criticality of securing secure accommodations and initiating legal safeguards at an early stage in the process.

Although innovative, this study has several methodological limitations. First, it is based on official data, analyzed through the lens of the criminal career paradigm, and the results of this study should be understood in this context. By definition, studies of criminal careers focus on crimes reported to the authorities and are subject to reliability limitations. Assessing the prevalence of sexual offending is particularly difficult as, although there is no precise estimate of the dark figure of crime in relation to such phenomena, we hypothesize that it is very high in an IPV context (see Bagwell-Gray et al., 2015). In line with several previous studies, the measurement and understanding of IPV are particularly challenging, with limited available information, especially regarding the most severe cases. Therefore, caution should be exercised when interpreting the results, as they are likely not representative of the overall distribution and characteristics of IPV but rather limited to cases reported to authorities. Thus we cannot know if the results of this study apply to all men who engage in IPSV. Nor can they be understood as providing an explanation of criminal phenomenon but rather suggest a way to focus on the official trajectory of criminal careers through use of a specific paradigm. Second, the study has a large sample size, making possible a more representative analysis of the phenomenon. Although this is a strength of our study, it is recognized that large numbers can lead to increased incidences of significance and that attention must be paid to effect sizes and confidence intervals to better understand the real impact of each indicator. Finally, while our study has focused on criminal and violent behaviors, it is important to acknowledge that research indicates the presence of specific criminogenic needs that may contribute to a continuous pattern of violence, particularly in cases of IPV involving both sexual and nonsexual violence. Unfortunately, it was not possible to incorporate such information into our analysis, which would have enriched the comprehensiveness of our study’s findings.

Future studies should replicate this analysis with other samples to determine the validity of the proposed trends and classifications. It would also be important to be able to associate the criminal trajectories with motivational and individual indicators to obtain a more accurate and effective overview of the phenomenon for practitioners. Finally, additional studies are needed to understand the differences and similarities between men who engage in IPSV and other individuals involved in sexual offenses. Such findings could lead to more tailored management and treatment of men who engage in IPSV.

## Supplemental Material

Supplemental Material - Violent Partners or a Specific Class of Offenders? A Criminal Career Approach to Understanding Men Involved in Intimate Partner Sexual Violence Supplemental Material for Violent Partners or a Specific Class of Offenders? A Criminal Career Approach to Understanding Men Involved in Intimate Partner Sexual Violence by Julien Chopin, Francis Fortin, Sarah Paquette, Jean-Pierre Guay, Olivier Péloquin and Eric Chartrand in Sexual Abuse

## Notes

1. We used the non-parametric Mann–Whitney *U* test because the distribution did not follow a normal distribution pattern.

2. Non-parametrical Kruskal–Wallis test was used as continuous variables did not follow a normal distribution.

## References

[bibr1-10790632231224356] Aikenhead M. (2018). Non-consensual disclosure of intimate images as a crime of gender-based violence. Canadian Journal of Women and the Law, 30(1), 117–143. 10.3138/cjwl.30.1.117

[bibr2-10790632231224356] Bachman R. Saltzman L. E. (1995). Violence against women: Estimates from the redesigned survey. US Department of Justice, Office of Justice Programs, Bureau of Justice.

[bibr3-10790632231224356] Bagwell-Gray M. E. (2021). Women’s experiences of sexual violence in intimate relationships: Applying a new taxonomy. Journal of Interpersonal Violence, 36(13–14), NP7813–NP7839. 10.1177/0886260519827667 30791809

[bibr4-10790632231224356] Bagwell-Gray M. E. Messing J. T. Baldwin-White A. (2015). Intimate partner sexual violence: A review of terms, definitions, and prevalence. Trauma, Violence, & Abuse, 16(3), 316–335. 10.1177/1524838014557290 25561088

[bibr5-10790632231224356] Barker L. C. Stewart D. E. Vigod S. N. (2019). Intimate partner sexual violence: An often overlooked problem. Journal of Women’s Health, 28(3), 363–374. 10.1089/jwh.2017.6811 30335574

[bibr6-10790632231224356] Basile K. C. (2002). Prevalence of wife rape and other intimate partner sexual coercion in a nationally representative sample of women. Violence & Victims, 17(5), 511–524. 10.1891/vivi.17.5.511.33717 12477095

[bibr7-10790632231224356] Beauregard E. DeLisi M. (2018). Stepping stones to sexual murder: The role of developmental factors in the etiology of sexual homicide. Journal of Criminal Psychology, 8(3), 199–214. 10.1108/JCP-02-2018-0010

[bibr8-10790632231224356] Beauregard E. DeLisi M. (2021). Unraveling the personality profile of the sexual murderer, Journal of Interpersonal Violence, 36(7–8), 3536–3556, Advanced online publication. 10.1177/0886260518777012 29783916

[bibr9-10790632231224356] Beauregard E. DeLisi M. Hewitt A. N. (2018). Sexual murderers: Sex offender, murderer, or both? Sexual Abuse: A Journal of Research and Treatment, 30(8), 932–950. 10.1177/1079063217711446 28583030

[bibr10-10790632231224356] Beauregard E. Martineau M. M. (2017). The sexual murderer: Offender behavior and implications for practice. Routledge.

[bibr11-10790632231224356] Bishop C. (2022). The impact of proposed intimate image abuse offences on domestic violence and abuse. Northern Ireland Legal Quarterly, 73(AD2), 125–153. 10.53386/nilq.v73iad2.969

[bibr12-10790632231224356] Black M. C. Basile K. C. Breiding M. J. Smith S. G. Walters M. L. Merrick M. T. Chen J. Stevens M. R. (2011). National intimate partner and sexual violence survey: 2010 summary report. https://www.cdc.gov/violenceprevention/pdf/nisvs_report2010-a.pdf

[bibr13-10790632231224356] Blosnich J. R. Bossarte R. M. (2009). Comparisons of intimate partner violence among partners in same-sex and opposite-sex relationships in the United States. American Journal of Public Health, 99(12), 2182–2184. 10.2105/AJPH.2008.139535 19834003 PMC2775776

[bibr14-10790632231224356] Blumstein A. Cohen J. Roth A. Visher A. (1986) Criminal careers and career criminals (Vol. 1). National Academy Press.

[bibr15-10790632231224356] Breiding M. Basile K. C. Smith S. G. Black M. C. Mahendra R. R. (2015). Intimate partner violence surveillance: Uniform definitions and recommended data elements. Version 2.0.

[bibr16-10790632231224356] Brownfield D. Sorenson A. M. (1987). A latent structure analysis of delinquency. Journal of Quantitative Criminology, 3(2), 103–124. 10.1007/BF01064211

[bibr17-10790632231224356] Campbell J. C. Alford P. (1989). The dark consequences of marital rape. American Journal of Nursing, 89(7), 946–949. 10.2307/3426372 2619795

[bibr18-10790632231224356] Cavanaugh C. E. Messing J. T. Del‐Colle M. O’Sullivan C. Campbell J. C. (2011). Prevalence and correlates of suicidal behavior among adult female victims of intimate partner violence. Suicide and Life-Threatening Behavior, 41(4), 372–383. 10.1111/j.1943-278X.2011.00035.x 21535096 PMC3152586

[bibr86-9107906322315] Chopin J. Fortin F. Guay J.-P. Péloquin O. Paquette S. Chartrand E. (2023). ‘Till death do us part’: An integrated multi-theoretical approach to identify predictors of intimate partner homicide. Journal of Criminal Justice, 88, 102101.

[bibr19-10790632231224356] Coley S. L. McCarthy R. J. Milner J. S. Ormsby L. Travis W. J. (2016). Intimate partner maltreatment recidivism in US air force families. Military Medicine, 181(8), 926–930. 10.7205/MILMED-D-15-00333 27483535

[bibr20-10790632231224356] Collins L. M. Lanza S. T. (2010). Latent class and latent transition analysis: With applications in the social, behavioral, and health sciences. Wiley.

[bibr21-10790632231224356] Creswell J. D. Creswell J. W. (2017). Research design: Qualitative, quantitative, and mixed methods approaches (3rd ed.). Sage.

[bibr22-10790632231224356] Cunha O. Pinheiro M. Gonçalves R. A. (2022). Intimate partner violence, psychopathy, and recidivism: Do psychopathic traits differentiate first-time offenders from repeated offenders? Victims and Offenders, 17(2), 199–218. 10.1080/15564886.2021.1885545

[bibr23-10790632231224356] DeLisi M. (2006). Zeroing in on early arrest onset: Results from a population of extreme career criminals. Journal of Criminal Justice, 34(1), 17–26. 10.1016/j.jcrimjus.2005.11.002

[bibr24-10790632231224356] DeLisi M. Beauregard E. (2018). Adverse childhood experiences and criminal extremity: New evidence for sexual homicide. Journal of Forensic Sciences, 63(2), 484–489. 10.1111/1556-4029.13584 28834569

[bibr25-10790632231224356] DeLisi M. Piquero A. R. (2011). New frontiers in criminal careers research, 2000–2011: A state-of-the-art review. Journal of Criminal Justice, 39(4), 289–301. 10.1016/j.jcrimjus.2011.05.001

[bibr26-10790632231224356] Dobash R. E. Dobash R. P. Cavanagh K. (2009). “Out of the blue” men who murder an intimate partner. Feminist Criminology, 4(3), 194–225. 10.1177/1557085109332668

[bibr27-10790632231224356] Dobash R. E. Dobash R. P. Cavanagh K. Medina-Ariza J. (2007). Lethal and nonlethal violence against an intimate female partner: Comparing male murderers to nonlethal abusers. Violence Against Women, 13(4), 329–353. 10.1177/1077801207299204 17420514

[bibr28-10790632231224356] Dowling C. Boxall H. Morgan A. (2021). The criminal career trajectories of domestic violence offenders. Trends and Issues in Crime and Criminal Justice No. 624, 1–17.

[bibr29-10790632231224356] Eaton A. A. Noori S. Bonomi A. Stephens D. P. Gillum T. L. (2021). Nonconsensual porn as a form of intimate partner violence: Using the power and control wheel to understand nonconsensual porn perpetration in intimate relationships. Trauma, Violence, & Abuse, 22(5), 1140–1154. 10.1177/1524838020906533 32100637

[bibr30-10790632231224356] Finkelhor D. Yllo K. (1985). License to rape: Sexual abuse of wives. Free Press.

[bibr31-10790632231224356] Goldstein D. A. Cantos A. L. Brenner L. H. Verborg R. J. Kosson D. S. (2016). Perpetrator type moderates the relationship between severity of intimate partner violence and recidivism. Criminal Justice and Behavior, 43(7), 879–898. 10.1177/0093854815616841

[bibr32-10790632231224356] Graham-Bermann S. A. DeVoe E. R. Mattis J. S. Lynch S. Thomas S. A. (2006). Ecological predictors of traumatic stress symptoms in Caucasian and ethnic minority children exposed to intimate partner violence. Violence Against Women, 12(7), 663–692. 10.1177/1077801206290216 16777951

[bibr33-10790632231224356] Guay J.-P. Fortin F. Chopin J. Peloquin O. Raiche A.-P. (2021). Bracelets anti-rapprochement: Entre prédiction et décision: Une approche basée sur les données probantes au sujet de l’application du bracelet anti-rapprochement en matière de violence conjugale en contexte québécois [Anti-Attack bracelets: Between prediction and decision: An evidence-based approach to the application of anti-attack bracelets to domestic violence in the Quebec context]. University of Montreal.

[bibr34-10790632231224356] Hilton N. Z. Eke A. W. (2016). Non-specialization of criminal careers among intimate partner violence offenders. Criminal Justice and Behavior, 43(10), 1347–1363. 10.1177/0093854816637886

[bibr35-10790632231224356] Holtzworth-Munroe A. Meehan J. C. Herron K. Rehman U. Stuart G. L. (2000). Testing the Holtzworth-Munroe and Stuart (1994) batterer typology. Journal of Consulting and Clinical Psychology, 68(6), 1000–1019. 10.1037//0022-006x.68.6.1000 11142534

[bibr36-10790632231224356] Holtzworth-Munroe A. Stuart G. L. (1994). Typologies of male batterers: Three subtypes and the differences among them. Psychological Bulletin, 116(3), 476–497. 10.1037/0033-2909.116.3.476 7809309

[bibr37-10790632231224356] Jones A. S. Gondolf E. W. (2002). Assessing the effect of batterer program completion on reassault: An instrumental variables analysis. Journal of Quantitative Criminology, 18(1), 71–98. 10.1023/a:1013244929733

[bibr38-10790632231224356] Jung S. Faitakis M. Cheema H. (2021). A comparative profile of intimate partner sexual violence. Journal of Sexual Aggression, 27(1), 95–105. 10.1080/13552600.2020.1722268

[bibr39-10790632231224356] Kivisto A. J. (2015). Male perpetrators of intimate partner homicide: A review and proposed typology. The journal of the American Academy of Psychiatry and the Law, 43(3), 300–312. 26438808

[bibr40-10790632231224356] Kyvsgaard B. (2002). The criminal career: The Danish longitudinal study. Cambridge University Press.

[bibr41-10790632231224356] Léveillée S. Vignola-Lévesque C. Brisson M. Champagne C. (2022). Enjeux psychosociaux des auteurs de violences conjugales sexuelles. Sexologies, 31(1), 14–26. 10.1016/j.sexol.2021.07.001

[bibr42-10790632231224356] Logan T. Cole J. R. Shannon L. A. (2007). A mixed-methods examination of sexual coercion and degradation among women in violent relationships who do and do not report forced sex. Violence & Victims, 22(1), 71–94. 10.1891/vv-v22i1a005 17390564

[bibr43-10790632231224356] Logan T. Walker R. Cole J. (2015). Silenced suffering: The need for a better understanding of partner sexual violence. Trauma, Violence, & Abuse, 16(2), 111–135. 10.1177/1524838013517560 24379191

[bibr44-10790632231224356] Loinaz I. (2014). Typologies, risk and recidivism in partner-violent men with the B-SAFER: A pilot study. Psychology, Crime and Law, 20(2), 183–198. 10.1080/1068316x.2013.770854

[bibr45-10790632231224356] Lucas K. T. (2022). Deepfakes and domestic violence: Perpetrating intimate partner abuse using video technology. Victims and Offenders, 17(5), 647–659. 10.1080/15564886.2022.2036656

[bibr46-10790632231224356] Mackay J. Bowen E. Walker K. O’Doherty L. (2018). Risk factors for female perpetrators of intimate partner violence within criminal justice settings: A systematic review. Aggression and Violent Behavior, 41, 128–146. 10.1016/j.avb.2018.06.004.

[bibr47-10790632231224356] MacLeod J. F. Grove P. Farrington D. (2012). Explaining criminal careers: Implications for justice policy. Oxford University Press.

[bibr48-10790632231224356] McFarlane J. Malecha A. Gist J. Watson K. Batten E. Hall I. Smith S. (2005). Intimate partner sexual assault against women and associated victim substance use, suicidality, and risk factors for femicide. Issues in Mental Health Nursing, 26(9), 953–967. 10.1080/01612840500248262 16203648

[bibr49-10790632231224356] Ménard K. S. Anderson A. L. Godboldt S. M. (2009). Gender differences in intimate partner recidivism: A 5-year follow-up. Criminal Justice and Behavior, 36(1), 61–76. 10.1177/0093854808325905

[bibr50-10790632231224356] Messing J. T. Campbell J. C. Snider C. (2017). Validation and adaptation of the danger assessment‐5: A brief intimate partner violence risk assessment. Journal of Advanced Nursing, 73(12), 3220–3230. 10.1111/jan.13459 28921610

[bibr51-10790632231224356] Messing J. T. Thaller J. Bagwell M. (2014). Factors related to sexual abuse and forced sex in a sample of women experiencing police-involved intimate partner violence. Health & Social Work, 39(3), 181–191. 10.1093/hsw/hlu026 25095631

[bibr52-10790632231224356] Morgan W. Gilchrist E. (2010). Risk assessment with intimate partner sex offenders. Journal of Sexual Aggression, 16(3), 361–372. 10.1080/13552600.2010.502976

[bibr53-10790632231224356] Murphy C. M. Musser P. H. Maton K. I. (1998). Coordinated community intervention for domestic abusers: Intervention system involvement and criminal recidivism. Journal of Family Violence, 13(3), 263–284. 10.1023/a:1022841022524

[bibr54-10790632231224356] Oram S. Fisher H. L. Minnis H. Seedat S. Walby S. Hegarty K. Rouf K. Angénieux C. Callard F. Chandra P. S. Fazel S. Garcia-Moreno C. Henderson M. Howarth E. MacMillan H. L. Murray L. K. Othman S. Robotham D. Rondon M. B. Howard L. M. (2022). The lancet psychiatry commission on intimate partner violence and mental health: Advancing mental health services, research, and policy. The Lancet Psychiatry, 9(6), 487–524. 10.1016/S2215-0366(22)00008-6 35569504

[bibr55-10790632231224356] Piquero A. R. Farrington D. P. Blumstein A. (2003). The criminal career paradigm. Crime and Justice, 30, 359–506. 10.1086/652234.

[bibr56-10790632231224356] Piquero A. R. Farrington D. P. Blumstein A. (2007). Key issues in criminal career research: New analyses of the Cambridge study in delinquent development. Cambridge University Press.

[bibr57-10790632231224356] Piquero A. R. Theobald D. Farrington D. P. (2014). The overlap between offending trajectories, criminal violence, and intimate partner violence. International Journal of Offender Therapy and Comparative Criminology, 58(3), 286–302. 10.1177/0306624X12472655 23315428

[bibr58-10790632231224356] Proulx J. Beauregard E. (2014). Pathways in the offending process of marital rapists. In Proulx J. Beauregard E. Lussier P. Leclerc B. (Eds.), Pathways to sexual aggression (p. 27). Routledge.

[bibr59-10790632231224356] Puri M. Tamang J. Shah I. (2011). Suffering in silence: Consequences of sexual violence within marriage among young women in Nepal. BMC Public Health, 11, 29–210. 10.1186/1471-2458-11-29. 21223603 PMC3091539

[bibr60-10790632231224356] Rempel M. Labriola M. Davis R. C. (2008). Does judicial monitoring deter domestic violence recidivism? Results of a quasi-experimental comparison in the bronx. Violence Against Women, 14(2), 185–207. 10.1177/1077801207312535 18212340

[bibr61-10790632231224356] Robinson A. L. (2017). Serial domestic abuse in wales: An exploratory study into its definition, prevalence, correlates, and management. Victims and Offenders, 12(5), 643–662. 10.1080/15564886.2016.1187691

[bibr62-10790632231224356] Rollè L. Giardina G. Caldarera A. M. Gerino E. Brustia P. (2018). When intimate partner violence meets same sex couples: A review of same sex intimate partner violence. Frontiers in Psychology, 9, 1506. 10.3389/fpsyg.2018.01506. 30186202 PMC6113571

[bibr63-10790632231224356] Russell D. E. (1990). Rape in marriage, exp. and rev. Indiana University Press.

[bibr64-10790632231224356] Schwartz G. (1978). Estimating the dimension of a model. Annals of Statistics, 6(2), 461–464. 10.1214/aos/1176344136

[bibr65-10790632231224356] Seyller M. Denis C. Dang C. Boraud C. Lepresle A. Lefèvre T. Chariot P. (2016). Intimate partner sexual assault: Traumatic injuries, psychological symptoms, and perceived social reactions. Obstetrics & Gynecology, 127(3), 516–526. 10.1097/AOG.0000000000001288 26855090

[bibr66-10790632231224356] Snider C. Webster D. O’Sullivan C. S. Campbell J. (2009). Intimate partner violence: Development of a brief risk assessment for the emergency department. Academic Emergency Medicine: Official Journal of the Society for Academic Emergency Medicine, 16(11), 1208–1216. 10.1111/j.1553-2712.2009.00457.x 20053241

[bibr67-10790632231224356] Sparks B. Wielinga F. Jung S. Olver M. E. (2020). Recidivism risk and criminogenic needs of individuals who perpetrated intimate partner sexual violence offenses. Sexual Offending: Theory, Research, and Prevention, 15, 1–20. 10.5964/sotrap.3713.

[bibr68-10790632231224356] Stefanska E. B. Beech A. Carter A. J. (2016). A systematic review of the literature comparing male non-serial sexual killers and sexual aggressors: Examining homogeneous and heterogeneous characteristics of these groups. Journal of Sexual Aggression, 22(3), 323–341. 10.1080/13552600.2015.1126657

[bibr69-10790632231224356] Symes L. Maddoux J. McFarlane J. Nava A. Gilroy H. (2014). Physical and sexual intimate partner violence, women’s health and children’s behavioural functioning: Entry analysis of a seven‐year prospective study. Journal of Clinical Nursing, 23(19–20), 2909–2918. 10.1111/jocn.12542 24443832

[bibr70-10790632231224356] Theobald D. Farrington D. P. Coid J. W. Piquero A. R. (2016). Are male perpetrators of intimate partner violence different from convicted violent offenders? Examination of psychopathic traits and life success in males from a community survey. Journal of Interpersonal Violence, 31(9), 1687–1718. 10.1177/0886260515569061 25681163

[bibr71-10790632231224356] Tjaden P. G. (2000). Extent, nature, and consequences of intimate partner violence. US Department of Justice, Office of Justice Programs, National Institute of Justice, 2000.

[bibr72-10790632231224356] Tjaden P. G. Thoennes N. (2006). Extent, nature, and consequences of rape victimization: Findings from the national violence against women survey. National Institute of Justice.

[bibr73-10790632231224356] Ullman S. E. Sigurvinsdottir R. (2015). Intimate partner violence and drinking among victims of adult sexual assault. Journal of Aggression, Maltreatment & Trauma, 24(2), 117–130. 10.1080/10926771.2015.996312 PMC4350239 25750512

[bibr74-10790632231224356] Vaughn M. G. DeLisi M. Beaver K. M. Howard M. O. (2008). Toward a quantitative typology of burglars: A latent profile analysis of career offenders. Journal of Forensic Sciences, 53(6), 1387–1392. 10.1111/j.1556-4029.2008.00873.x 18798769

[bibr87-9107906322314] Wallace M (2009). La mesure de la criminalité au Canada : présentation de l’Indice de gravité de la criminalité et des améliorations au Programme de déclaration uniforme de la criminalité [Measuring crime in Canada: Introducing the Crime Severity Index and improvements to the Uniform Crime Reporting Survey]. Statistics Canada.

[bibr75-10790632231224356] Weaver T. L. Allen J. A. Hopper E. Maglione M. L. McLaughlin D. McCullough M. A. Jackson M. K. Brewer T. (2007). Mediators of suicidal ideation within a sheltered sample of raped and battered women. Health Care for Women International, 28(5), 478–489. 10.1080/07399330701226453 17469001

[bibr76-10790632231224356] Weldon S. (2016). Implicit theories in intimate partner violence sex offenders: An interpretative phenomenological analysis. Journal of Family Violence, 31(3), 289–302. 10.1007/s10896-015-9774-y

[bibr77-10790632231224356] White S. J. Sin J. Sweeney A. Salisbury T. Wahlich C. Montesinos Guevara C. M. Gillard S. Brett E. Allwright L. Iqbal N. (2023). Global prevalence and mental health outcomes of intimate partner violence among women: A systematic review and meta-analysis. Trauma, Violence, and Abuse, 25(1), 15248380231155529. 10.1177/152483802311555 PMC10666489 36825800

[bibr78-10790632231224356] Williams K. S. Bierie D. M. (2015). An incident-based comparison of female and male sexual offenders. Sexual Abuse: A Journal of Research and Treatment, 27(3), 235–257. 10.1177/1079063214544333 25079779

[bibr79-10790632231224356] Woodin E. M. O’Leary K. D. (2006). Partner aggression severity as a risk marker for male and female violence recidivism. Journal of Marital and Family Therapy, 32(3), 283–296. 10.1111/j.1752-0606.2006.tb01607.x 16933434

[bibr80-10790632231224356] Wooldredge J. (2007). Convicting and incarcerating felony offenders of intimate assault and the odds of new assault charges. Journal of Criminal Justice, 35(4), 379–389. 10.1016/j.jcrimjus.2007.05.003

[bibr81-10790632231224356] World Health Organization . (2012). Understanding and addressing violence against women: Intimate partner violence. https://www.who.int/publications/i/item/WHO-RHR-12.43

[bibr82-10790632231224356] Zara G. Farrington D. P. (2010). A longitudinal analysis of early risk factors for adult‐onset offending: What predicts a delayed criminal career? Criminal Behaviour and Mental Health: CB, 20(4), 257–273. 10.1002/cbm.763 20306484

[bibr83-10790632231224356] Zara G. Farrington D. P. (2013). Assessment of risk for juvenile compared with adult criminal onset implications for policy, prevention, and intervention. Psychology, Public Policy, and Law, 19(2), 235–249. 10.1037/a0029050

[bibr84-10790632231224356] Zara G. Farrington D. P. (2016). Criminal recidivism: Explanation, prediction and prevention. Routledge.

[bibr85-10790632231224356] Zara G. Gino S. Veggi S. Freilone F. (2022). Sexual femicide, non-sexual femicide and rape: Where do the differences lie? A continuum in a pattern of violence against women. Frontiers in Psychology, 13, 957327. 10.3389/fpsyg.2022.957327. 36389581 PMC9664082

