# Supplementary material for: Violent Partners or a Specific Class of Offenders? A Criminal Career Approach to Understanding Men Involved in Intimate Partner Sexual Violence
Source: Sex Abuse. 2023 Dec 27;37(2):153–80. doi: 10.1177/10790632231224356 (PMC11731407; doi:10.1177/10790632231224356)
Supplement: Supplemental Material - Violent Partners or a Specific Class of Offenders? A Criminal Career Approach to Understanding Men Involved in Intimate Partner Sexual Violence [file sj-pdf-1-sax-10.1177_10790632231224356.pdf]

Appendix

Appendix 1. Multicollinearity diagnosis (N=44,932)

	Tolerance	VIF
Number of homicides	0.82	1.23
Number of liberty violation offences	0.87	1.15
Number of violent offences	0.62	1.62
Number of property offences	0.59	1.70
Number of condition breaches	0.39	2.56
Number of drug offences	0.68	1.46
Number of sexual offences (non-domestic context)	0.97	1.04
Number of other offences	0.60	1.68
Variety	0.50	1.99
Lambda	0.74	1.35
Average gravity score	0.81	1.24
Age at the first criminal event	0.89	1.13

Appendix 2. ROC curve of binary logistic regression model (N=44,932)

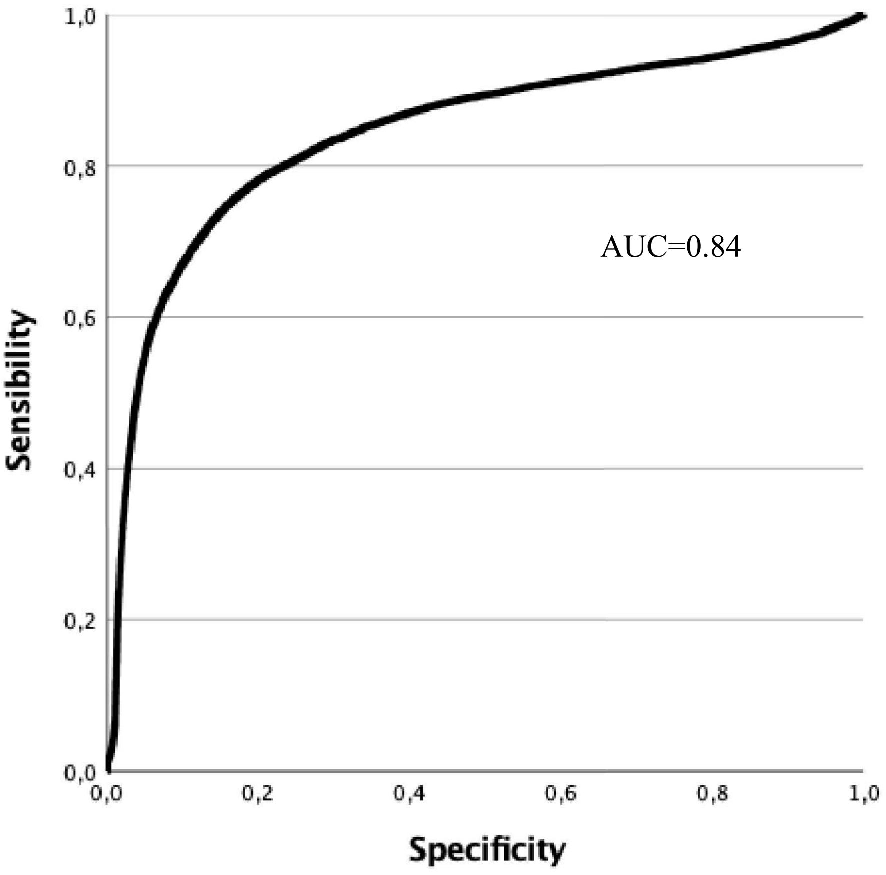

Appendix 3. Fit indices for latent profiles (N=44,932)

**Nb of profiles**	**LL**	**BIC(LL)**	**AIC(LL)**	**Adjusted BIC**	**VLMR**	**p-value**	**Entropy R^2**
1-Cluster	-156355.61	312881.16	312747.23	312823.96			1.00
2-Cluster	48747.67	-97146.02	-97421.34	-97263.60	410206.57	< 0.001	0.99
3-Cluster	134664.37	-268800.05	-269216.75	-268978.01	171833.41	< 0.001	0.99
**4-Cluster**	**200837.61**	**-400429.00**	**-401411.22**	**-400848.48**	**108911.83**	< 0.001	**0.99**
5-Cluster	185404.29	-369921.12	-370620.58	-370219.84	55564.43	< 0.001	0.99
6-Cluster	173055.39	-345043.95	-345884.78	-345403.05	-24697.79	2.06	0.98
7-Cluster	130948.37	-261188.66	-261746.74	-261427.01	-7432.00	1.00	0.98

Note: Boldface type indicates the selected model

Vuong-Lo-Mendel-Rubin likelihood ratio test not applicable for one-class model
